# Supplementary material for: Assessment of the Plans to Optimize Antimicrobial Use in the Pediatric Population in Catalan Hospitals: The VINCat Pediatric PROA SHARP Survey
Source: Antibiotics (Basel). 2023 Jan 26;12(2):250. doi: 10.3390/antibiotics12020250 (PMC9952038; doi:10.3390/antibiotics12020250)
Supplement: Supplementary file 1 [file antibiotics-12-00250-s001.zip › antibiotics-2123508-supplementary.pdf]

Supplementary Materials: members of VINCat pediatric PROA group

Berta Pujol-Soler. Department of Paediatrics. Hospital General de Granollers. Granollers; Spain.
Olga Martínez. Department of Paediatrics. Centro Médico Teknon. Grupo QuironSalud. Barcelona; Spain.
Eduard Solé Mir. Department of Paediatrics. Hospital Universitario Arnau de Vilanova de Lleida. Lleida; Spain.
Edurne Fernandez de Gamarra-Martínez. Department of Pharmacy. Hospital de la Santa Creu i Sant Pau. Barcelona; Spain.
Francesc Ripoll Oliveras. Pediatric Infectious Diseases Unit ICS-IAS. Department of Paediatrics. Hospital Santa Caterina. Girona; Spain.
Grisel Vilagrasa. Department of Paediatrics. Hospital Universitario Quiron Dexeus. Barcelona; Spain.
Isabel Vives Oñós. Department of Paediatrics. Hospital QuirónSalud Barcelona. Barcelona; Spain.
Isabel Zambudio. Department of Paediatrics. Hospital de Igualada. Igualada; Spain.
Javier Cantero Garcia. Department of Paediatrics. Hospital Comarcal de Blanes. Blanes; Spain.
Joan Manuel Torres Simón. Department of Paediatrics. Hospital de Palamós. Palamós; Spain.
José María Valle-T-Figueras. Department of Paediatrics. Hospital de la Santa Creu i Sant Pau. Barcelona; Spain.
Lourdes Garcia Rodríguez. Department of Paediatrics. Hospital de Mataró. Mataró; Spain.
Marcelina Algar Serrano, Department of Paediatrics. Hospital de Figueres. Figueres; Spain.
María José Elizari. Department of Paediatrics. Hospital de Barcelona. Barcelona; Spain.
Marina Fenoy Alejandre. Department of Paediatrics. Consorci Sanitari de Terrassa. Terrassa; Spain.
Marlene Alvarez. Department of Pharmacy. Hospital Universitario Germans Trias i Pujol de Badalona. Badalona; Spain.
María Méndez. Department of Paediatrics. Hospital Universitario Germans Trias i Pujol de Badalona. Badalona; Spain.
Montserrat Rodríguez-Reyes. Department of Pharmacy. Hospital Clínic de Barcelona (Seu Maternitat); Barcelona; Spain.
Montserrat Ruiz García, Department of Paediatrics. Hospital Universitario de Vic. Vic; Spain.
Neus Rius Gordillo. Department of Paediatrics. Hospital Universitario Sant Joan de Reus. Reus; Spain.
Núria López Segura. Department of Paediatrics. Hospital del Mar—Parc de Salut del Mar. Barcelona; Spain.
Núria Sanmartí Martínez, Department of Pharmacy. Hospital Universitario Mútua de Terrassa. Terrassa; Spain.
Olga Calavia. Department of Paediatrics. Hospital Universitario Joan XXIII de Tarragona. Tarragona; Spain.
Pilar Marcos. Department of Pharmacy., Hospital Universitario General de Catalunya. Sant. Joan Despí; Spain.
Raquel Aguilar Salmerón. Department of Pharmacy. Hospital Universitario de Girona Dr. Josep Trueta. Girona; Spain.
